# Enterohaemorrhagic *Escherichia coli* O121:H19 acquired an extended-spectrum *β*-lactamase gene during the development of an outbreak in two nurseries

**DOI:** 10.1099/mgen.0.000278

**Published:** 2019-06-19

**Authors:** Koji Kikuchi, Kenichi Lee, Hiroyuki Ueno, Kentaro Tomari, Sumie Kobori, Akihiko Kaetsu, Mari Matsui, Satowa Suzuki, Tsuyoshi Sekizuka, Makoto Kuroda, Motonobu Miyazaki, Makoto Ohnishi

**Affiliations:** ^1^ Saitama City Institute of Health Science and Research, Saitama, Japan; ^2^ Department of Bacteriology I, National Institute of Infectious Diseases, Tokyo, Japan; ^3^ Saitama City Health Center, Saitama, Japan; ^4^ Antimicrobial Resistance Research Center, National Institute of Infectious Diseases, Tokyo, Japan; ^5^ Pathogen Genomics Center, National Institute of Infectious Diseases, Tokyo, Japan

**Keywords:** enterohaemorrhagic *Escherichia coli*, extended spectrum *β*-lactamase, antimicrobial resistance, plasmid transfer, whole-genome sequence analysis

## Abstract

Enterohaemorrhagic *Escherichia coli* (EHEC) is an important human pathogen worldwide. Although serotype O157 is currently the most dominant and important EHEC strain, serotypes O26, O111, O91, O103 and O121 are also recognized as serious pathogens that affect public health. EHEC outbreaks often occur in nurseries and elderly care facilities. In 2012, a nursery outbreak of EHEC O121 occurred during which the bacterium acquired a plasmid-borne extended-spectrum *β*-lactamase (ESBL) gene. ESBL-producing *E. coli* O86 was concurrently isolated from one of the EHEC patients. Therefore, we investigated the isolates by whole-genome sequence (WGS) analysis to elucidate the transmission dynamics of the EHEC strains and the ESBL plasmid. According to WGS-based phylogeny, all 17 EHEC O121 isolates were clonal, while *E. coli* O86 was genetically distant from the EHEC O121 isolates. The complete sequence of an ESBL plasmid encoding the CTX-M-55 *β*-lactamase was determined using S1-PFGE bands, and subsequent mapping of the WGS reads confirmed that the plasmid sequences from EHEC O121 and *E. coli* O86 were identical. Furthermore, conjugation experiments showed that the plasmid was capable of conjugative transfer. These results support the hypothesis that EHEC O121 acquired an ESBL-producing plasmid from *E. coli* O86 during the outbreak. This report demonstrates the importance of implementing preventive measures during EHEC outbreaks to control both secondary infection and the spread of antimicrobial resistance factors.

## Data Summary

Genomic data have been deposited in GenBank (accession number: PRJDB7237).

**Outcome**

Our results demonstrate the importance of monitoring antimicrobial resistance during EHEC outbreaks. Local healthcare authorities, such as health centres, should take measures to prevent the spread not only of secondary infections, but also of antimicrobial resistance. Such measures could be carried out in cooperation with the medical and agricultural sectors, where intensive monitoring and control measures have been implemented for the monitoring of antimicrobial-resistant bacteria.

## Introduction

Enterohaemorrhagic *Escherichia coli* (EHEC) is an important human pathogen worldwide. EHEC produces Shiga toxin (Stx), and infections with EHEC strains present clinical symptoms such as diarrhoea, haemorrhagic colitis and life-threatening haemolytic uraemic syndrome (HUS) [1]. EHEC infections are commonly associated with contaminated food or direct contact with ruminants [2, 3]. In Japan, more than 3000 cases of EHEC infection are reported annually [4]. If necessary, EHEC isolates are collected in the National Institute of Infectious Diseases, Japan, and nation-wide surveillance of the bacterium has been performed using multilocus variable-number tandem-repeat analysis for serogroups O157, O26, O111, O91, O103, O121, O145 and O165, and pulsed-field gel electrophoresis (PFGE) for the other serogroups [4, 5]. Although serogroup O157 is currently the most dominant serotype in Japan, several reports have described nursery outbreaks by non-O157 EHEC [6–11]. EHEC infections often spread via secondary infection, probably due to EHEC’s low infectious dose. Young children and elderly people are more likely to suffer severe symptoms due to infection. Hence, the control and prevention of EHEC infection in facilities caring for these populations are important for public health.

In 2012, a nursery outbreak of EHEC O121 occurred during which the bacterium acquired antimicrobial resistance [12]. The isolates showed similar PFGE profiles. However, PFGE has limited typing resolution in some isolates, especially in EHEC O121 [13]. Therefore, we investigated the isolates in more detail by whole-genome sequence (WGS) analysis, which has been widely implemented in EHEC surveillance [6, 8, 14]. High-resolution phylogenetic analysis and the complete sequence of an extended-spectrum β-lactamase (ESBL) plasmid provided direct evidence of plasmid transfer from a non-toxigenic *E. coli* strain to an EHEC strain.

## Methods

### Outbreak information

**Fig. 1.**
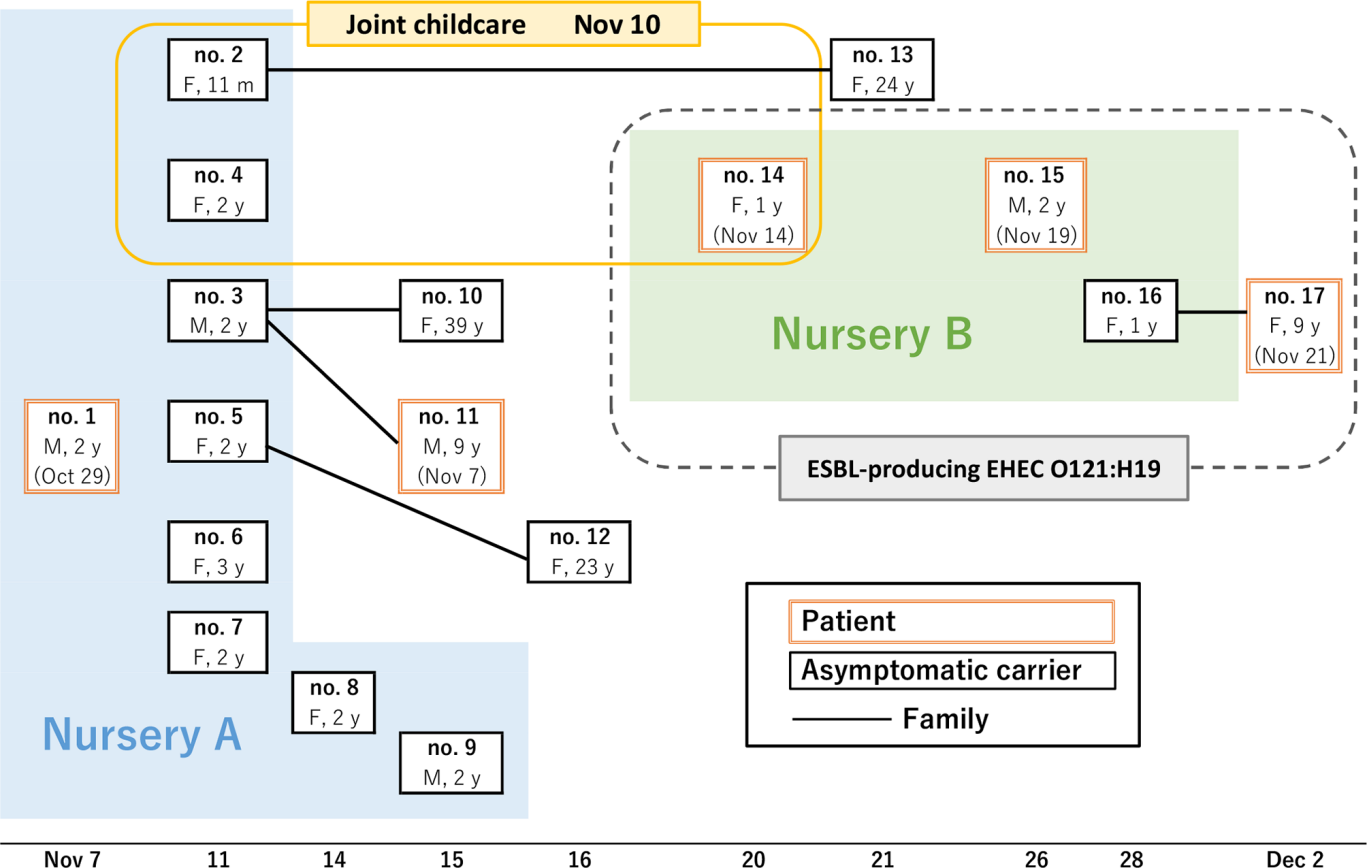
Overview of the outbreak in nurseries A and B. Each square represents one patient. Patient number, gender and age are shown in the squares (M, male; F, female; y, age in years). The X-axis represents the isolation date of each isolate. The onset dates are shown in parentheses. Children who attended the joint childcare are shown in a yellow box. Patients from whom ESBL-producing EHEC O121 were isolated were shown in a dotted line box.

We experienced an EHEC outbreak in two nurseries in November 2012 [12]. The relationships between the EHEC patients in the two nurseries are shown in Fig. 1. Initially, EHEC was isolated from a 2-year-old boy who had diarrhoea (patient 1). This isolate was immediately transported from hospital Z to Saitama City Institute of Health Science and Research, where it was examined to confirm its characteristics. Because he had been regularly attending nursery A, Saitama City Health Center investigated his family and nursery A to find the source of the infection. Forty-three faecal samples collected from subjects who had close contact with him in nursery A were tested and it was subsequently confirmed that eight children aged 9–39 months were asymptomatically infected with EHEC (patients 2–9). Next, 86 faecal samples from members of the patients’ families were tested and it was confirmed that 4 family members, the mothers of patients 2 and 5 and the mother and a brother of patient 3, were infected with EHEC (patients 10–13). One child presenting with diarrhoea (patient 11) was attending primary school K, but secondary infection was not found after subsequent investigation. During the investigation, EHEC was isolated from a 1-year-old girl attending nursery B who had bloody diarrhoea and a fever (patient 14). This isolate was immediately transported from hospital Y to Saitama City Institute of Health Science and Research and examined to confirm its characteristics. During the investigation by the Health Center, it was established that nursery A had a joint childcare period with nursery B during this outbreak. It was confirmed that several children, including patient 14 and two other EHEC patients in nursery A (patients 2 and 4), were involved in joint childcare. Because of this connection, the investigation was extended to include faecal samples collected from family members of patient 4 and people who attended the joint childcare in nurseries A and B. As a result, it was subsequently confirmed that two children (aged 20–29 months) were infected with EHEC (patients 15 and 16). These patients had diarrhoea after joint childcare. During further testing of faecal samples from members of their families, one case of diarrhoea was confirmed in a student from primary school L (patient 17), where no other secondary infection was found during the investigation. The investigation continued for almost 3 months until all the faecal samples from the EHEC-positive patients were negative for EHEC.

In total, 5 patients and 12 asymptomatic carriers were confirmed in this outbreak. No subjects presented HUS in the outbreak. We attempted to identify the source and the route of EHEC infection in the nurseries and families; however, the source of this EHEC outbreak remains unknown.

In our investigation, the antimicrobial resistance profiles of the EHEC O121 isolates from nurseries A and B differed, although antimicrobials were not used in all the patients and carriers in this outbreak. All of the EHEC O121 isolates from nursery B were ESBL producers, while the isolates from nursery A were not (as described in the Results section). Furthermore, ESBL-producing *E. coli* O86 was concurrently isolated in hospital Y from one of the patients in nursery B (no. 14). ESBL-producing *E. coli* had been routinely examined in hospital Y using a microbiological automatic analyser in the same way as for EHEC detection, although none of the samples from the other EHEC patients in the nurseries were examined for ESBL-producing *Enterobacteriaceae *. These results suggested the transfer of an ESBL plasmid, which prompted us to further characterize the isolates using WGS analyses.

### Isolates used in this study

Information on the isolates used in this study is provided in Table 1. We used DHL (Eiken Chemical Co. Ltd, Tokyo, Japan) and CHROMagar STEC (Kanto Chemical Co., Tokyo, Japan) as non-selective and selective agar plates, respectively, to isolate the EHEC strains in this outbreak. Molecular biological screening of the EHEC isolates using a colony sweep method and PCR was performed as described previously [15]. Briefly, colony sweeps from DHL agar plates were suspended in distilled water and subsequently subjected to boiling for 10 min for DNA extraction. The extracted DNA was examined for the presence of *stx* genes using PCR. Single colonies of s*tx* gene-positive samples were obtained using selective agar plates and then examined using biochemical tests and serotyping. The biochemical tests were performed using triple-sugar iron slants (Nissui Pharmaceutical Co., Tokyo, Japan), lysine indole motility medium (Eiken Chemical Co. Ltd), Simmons citrate agar slants (Eiken Chemical Co. Ltd) and API20E (bioMerieux Japan LTD., Tokyo, Japan). Serotyping was performed using agglutination tests with the *E. coli* antisera Seiken Set 1 (Denka Seiken Co., Tokyo, Japan) for the O antigen and Set 2 (Denka Seiken) for the H antigen, according to the manufacturer’s instructions. The presence of virulence factors such as *stx1*-, *stx2*- and *eae*-encoded bacterial intimate adherence factor, and *hlyA*-encoded enterohaemolysin, was determined by PCR [15, 16].

**Table 1.** Characteristics of isolates obtained from the nursery outbreak

Isolate ID	Patient*	Nursery school†	Phylogenetic group	Serotype	MLST	rMLST
EC12035	1	A	B1	O121:H19	655	15 635
EC12036	2	A	B1	O121:H19	655	15 635
EC12037	3	A	B1	O121:H19	655	15 635
EC12038	4	A	B1	O121:H19	655	New
EC12039	5	A	B1	O121:H19	655	15 635
EC12040	6	A	B1	O121:H19	655	15 635
EC12041	7	A	B1	O121:H19	655	15 635
EC12042	8	A	B1	O121:H19	655	15 635
EC12043	9	A	B1	O121:H19	655	15 635
EC12044	10	A family	B1	O121:H19	655	15 635
EC12045	11	A family	B1	O121:H19	655	15 635
EC12046	12	A family	B1	O121:H19	655	15 635
EC12049	13	A family	B1	O121:H19	655	15 635
EC12050	14	B	B1	O121:H19	655	15 635
EC12051	14	B	E	O86:H2	349	15 101
EC12052	15	B	B1	O121:H19	655	15 635
EC12053	16	B	B1	O121:H19	655	15 635
EC12054	17	B family	B1	O121:H19	655	15 635

*The numbers correspond to those in a previous report [12].

†Nursery school where the patient attended. Family member of nursery school attendee is also shown.

### Antimicrobial susceptibility testing and identification of the ESBL

The antimicrobial susceptibility of each isolate was examined using the Kirby–Bauer disk diffusion method according to the procedures and criteria of the Clinical Laboratory Standards Institute [17]. The disk diffusion method was performed on Mueller–Hinton II agar plates (Becton Dickinson, Franklin Lakes, NJ, USA) with the following antimicrobial agents: ampicillin, cefotaxime, kanamycin, gentamicin, streptomycin, tetracycline, chloramphenicol, ciprofloxacin, norfloxacin, nalidixic acid, fosfomycin (50 µg) and sulfamethoxazole/trimethoprim (Becton Dickinson). For the isolates showing cefotaxime resistance, the minimum inhibitory concentrations (MICs) of the *β*-lactam antibiotics were examined via a broth microdilution method using a Dry Plate (Eiken Chemical Co. Ltd). The antimicrobials used for the MIC measurements were as follows: ampicillin, cefotaxime, ceftazidime, cefpodoxime, cefepime, imipenem, meropenem and aztreonam. The production of ESBL was confirmed by the disk diffusion method using cefotaxime, ceftazidime or cefpodoxime and clavulanic acid (Eiken Chemical Co. Ltd). The presence of the ESBL genes was confirmed by PCR and sequencing of the CTX-M-type genes established previously [18, 19]. PCR products were electrophoresed in 2.0 % SeaKem ME agarose (Lonza Japan Co., Tokyo, Japan) and subsequently purified with the MinElute Gel Extraction kit (Qiagen, Venlo, Netherlands). The purified DNA was sequenced with a BigDye Terminator v3.1 Cycle Sequencing kit and a model 3130 DNA sequencer (Thermo Fisher Scientific, Inc., Waltham, MA, USA).

### Phylogenetic analysis and isolate characterization using WGS

WGS was obtained from EHEC O121 and *E. coli* O86 isolates and the phylogenetic relationship was inferred with 43 strains of EHEC and non-EHEC *E. coli* data on public database (Table S1, available in the online version of this article). Short-read sequences of the EHEC O121 and *E. coli* O86 isolates were obtained using MiSeq (Illumina, San Diego, CA, USA). The genomic DNA librariere prepared using a Nextera XT DNA sample prep kit (Illumina). The pooled libraries were subjected to multiplexed paired-end sequencing (300 mer×2). Single-nucleotide polymorphisms (SNPs) were identified by a mapping-based method. Short reads were mapped to the genome of EHEC O26:H11 11368 (NCBI accession no. AP010953) as a reference sequence using bwa v.0.7.17 [20] and SNPs were identified using samtools v.1.7 [21] and VarScan v.2.4.3 [22] software. Exact and inexact repetitive regions longer than 50 bp were detected using the nucmer, repeat-match and exact-tandems functions of MUMmer v.3.2259 [23] and were removed for further analyses. SNP clusters (>two SNPs within 100 bp) were removed to exclude any SNPs in recombinogenic regions. Phylogenetic relationships were determined by reconstructing a phylogenetic tree using the maximum-likelihood method based on the GTR-GAMMA model with 1000 bootstraps using RAxML v.7.2.860 [24]. For the higher resolution analysis, SNPs were only identified in outbreak-related O121 isolates using the E12035 draft genome as a reference and a minimum spanning tree was constructed using GrapeTree software [25]. The short reads were assembled using SPAdes v.3.11.1 with the ‘--careful’ option [26]. Contigs of each isolate were characterized by public databases as follows: serotype (SerotypeFinder 1.1: https://cge.cbs.dtu.dk/services/SerotypeFinder/), phylogenetic group [27], presence of virulence genes (VirulenceFinder 1.5: https://cge.cbs.dtu.dk/services/VirulenceFinder/), antimicrobial resistance genes (ResFinder 3.0: https://cge.cbs.dtu.dk/services/ResFinder/) and multilocus sequence typing (MLST) (EnteroBase: http://enterobase.warwick.ac.uk/).

### Determining plasmid sequences

To determine the sequence of the plasmid encoding the ESBL, S1-PFGE was performed using a single-strand nuclease, S1 nuclease [28]. ESBL-producing EHEC O121 (EC12050) and *E. coli* O86 (EC12051) isolates were used for this analysis. S1-PFGE plugs for the ESBL-producing isolates were prepared using a 50-well Disposable Plug Mold (Bio-Rad Laboratories, Inc., Hercules, CA, USA). The plugs of genomic DNA were incubated at 35 °C for 40 min with 30 units of S1 nuclease (TaKaRa Bio Co., Shiga, Japan). The digested DNA was electrophoresed in 1.0 % Pulsed Field Certified Agarose (Bio-Rad Laboratories Inc.) in 0.5× TBE buffer with the running conditions generated by the auto-algorithm function of the CHEF Mapper XA system for 20 kbp to 400 kbp (Bio-Rad Laboratories, Inc.). The gel was stained with SYBR Gold nucleic acid gel stain (Life Technologies Co., OR, USA) and visualized using a blue LED transilluminator. The DNA bands predicted to be plasmids were excised, and the DNA was purified using the Zymoclean Large Fragment DNA Recovery kit (Funakoshi Co. Ltd., Tokyo, Japan). The DNA was subjected to MiSeq sequencing as described above. The obtained short reads were assembled by SPAdes. Because the ESBL plasmid from EHEC O121 has a shufflon region, which is difficult to assemble using Illumina short reads [29], TA cloning was performed using TArget Clone (Toyobo, Osaka, Japan) to determine the complete plasmid sequence. One of the cloned plasmids was sequenced via the dye terminator method as described above. Annotation of the plasmid was performed by DFAST [30].

### Conjugation assay of antimicrobial resistance plasmids

Transconjugation experiments for transferable plasmids were performed as described elsewhere, with minor modifications [31, 32]. Briefly, ESBL-producing *E. coli* O86 (EC12051), which is susceptible to rifampicin, was used as a donor, and *E. coli* DH5α (TaKaRa Bio Co.), which is resistant to rifampicin, was used as a recipient. Based on the WGS analyses, *E. coli* O86 was thought to possess two antimicrobial resistance plasmids, each of which harbours *bla*_CTX-M-55_ and *bla*_TEM-1C_. The plasmid harbouring *bla*_TEM-1C_ also harboured genes for aminoglycoside and sulfonamide resistance, as described in the Results section. Therefore, cefotaxime and streptomycin were used as selective agents for the plasmids harbouring *bla*_CTX-M-55_ and *bla*_TEM-1C_, respectively. The donor strain and DH5α were incubated for 2 hours at 37 °C and pour-plated onto LB agar plates containing 16 µg ml^−1^ of cefotaxime (FUJIFILM Wako Pure Chemical Co., Osaka, Japan) or 16 µg ml^−1^ of streptomycin (FUJIFILM Wako Pure Chemical Co.) and 50 µg ml^−1^ of rifampicin (FUJIFILM Wako Pure Chemical Co.). Colonies that acquired resistance to both cefotaxime or streptomycin and rifampicin were isolated as transconjugants. The frequency of transfer was calculated as the number of colony-forming units (c.f.u.) on the LB plates containing cefotaxime and rifampicin divided by the number of c.f.u. on the plates containing only cefotaxime. Susceptibility tests to the antimicrobials were performed as described above.

## Results

### Isolation of EHEC O121

A total of 17 EHEC-infected people were confirmed in two nurseries (Fig. 1 and Table 1). The serotype of the EHEC isolates was O121. All of the isolates carried *stx2*, *eae* and *hlyA*, according to PCR. *E. coli* O86 was concurrently detected in one of the EHEC O121 patients (patient 14). As far as we could determine, EHEC shedding lasted at least 33 days in the longest case (patient 5). Faecal samples from this patient returned to negative for EHEC 58 days after the first isolation.

### Antimicrobial susceptibilities and ESBL gene analysis

According to the disk diffusion test, none of the EHEC O121 isolates from nursery A showed resistance to the antimicrobials used in this study, while the EHEC O121 isolates from nursery B and the *E. coli* O86 isolate (EC12050-4) were resistant to ampicillin, cefotaxime, cefpodoxime and ceftazidime. All of the isolates were susceptible to the other antimicrobials, except for the *E. coli* O86 isolate, which was resistant to streptomycin. Further characterization of β-lactam resistance is shown in Table 2. The EHEC O121 isolates from nursery B (EC12050 and EC12052-4) and the *E. coli* O86 isolate were resistant to ampicillin, cefotaxime, ceftazidime, cefpodoxime, cefepime and aztreonam, but were susceptible to imipenem and meropenem according to their MICs. Furthermore, the resistance phenotypes of these five isolates were inhibited by clavulanic acid, suggesting ESBL production. A CTX-M-1-type gene was amplified by PCR in the ESBL producers. The sequences of the CTX-M-1-type genes were 100 % identical to *bla*_CTX-M-55_ of *E. coli* (NCBI accession no. LC095456). In the *E. coli* O86 isolate carrying *bla*_CTX-M-55_, *bla*_TEM_ was also detected by PCR.

**Table 2.** Susceptibility to β-lactams and sequence typesof β-lactamase gene of *Escherichia coli* isolates,and transconjugants in this outbreak

Patient	Isolate ID	Serotype	MIC (μg ml^−^^1^)								Inhibition with clavulanic acid	*bla* type
			ABPC*	CTX	CAZ	CPDX	CFPM	IPM	MEPM	AZT		
1	EC12035	O121:H19	2	≤0.25	0.12	0.25	≤0.25	0.06	≤0.03	≤0.06	nt†	na
2	EC12036	O121:H19	4	≤0.25	0.12	0.5	≤0.25	0.12	≤0.03	≤0.06	nt	na
3	EC12037	O121:H19	2	≤0.25	0.12	0.5	≤0.25	≤0.03	≤0.03	≤0.06	nt	na
4	EC12038	O121:H19	2	≤0.25	0.25	0.5	≤0.25	0.06	≤0.03	0.12	nt	na
5	EC12039	O121:H19	4	≤0.25	0.12	0.5	≤0.25	≤0.03	≤0.03	0.12	nt	na
6	EC12040	O121:H19	2	≤0.25	0.25	0.25	≤0.25	≤0.03	≤0.03	≤0.06	nt	na
7	EC12041	O121:H19	2	≤0.25	0.12	0.5	≤0.25	≤0.03	≤0.03	≤0.06	nt	na
8	EC12042	O121:H19	4	≤0.25	0.25	0.5	≤0.25	0.06	≤0.03	≤0.06	nt	na
9	EC12043	O121:H19	2	≤0.25	0.12	0.5	≤0.25	0.06	≤0.03	≤0.06	nt	na
10	EC12044	O121:H19	2	≤0.25	0.25	0.25	≤0.25	0.06	≤0.03	≤0.06	nt	na
11	EC12045	O121:H19	2	≤0.25	0.25	0.25	≤0.25	≤0.03	≤0.03	≤0.06	nt	na
12	EC12046	O121:H19	2	≤0.25	0.12	0.25	≤0.25	≤0.03	≤0.03	≤0.06	nt	na
13	EC12049	O121:H19	2	≤0.25	0.12	0.25	≤0.25	0.06	≤0.03	≤0.06	nt	na
14	EC12051	O86:H2	>512	32	8	128	8	0.06	≤0.03	16	+	CTX-M-55
	EC12050	O121:H19	>512	64	8	>256	16	0.06	≤0.03	32	+	CTX-M-55
15	EC12052	O121:H19	>512	64	8	256	16	0.06	≤0.03	16	+	CTX-M-55
16	EC12053	O121:H19	>512	32	4	256	16	0.06	≤0.03	16	+	CTX-M-55
17	EC12054	O121:H19	>512	32	8	256	8	0.06	≤0.03	32	+	CTX-M-55
na	DH5α	O16:H48	2	≤0.25	0.06	0.5	≤0.25	0.12	≤0.03	≤0.06	na	–
na	Transconjugant (CTX)‡	O16:H48	>512	64	8	>256	8	0.25	≤0.03	32	+	CTX-M-55
na	Transconjugant (SM)§	O16:H48	>256	≤0.25	≤0.25	≤0.5	≤0.25	≤0.25	≤0.25	≤0.25	+	TEM-1c

*ABPC, ampicillin; CTX, cefotaxime; CAZ, ceftazidime; CPDX, cefpodoxime; CFPM, cefepime; IPM, imipenem; MEPM, meropenem; AZT, aztreonam

†+, presence; -, absence; nt, not tested; na, not applicable

‡Transconjugant obtained from conjugation assay using cefotaximeand rifampicin

§Transconjugant obtained from conjugation assay using streptomycin and rifampicin

### WGS analyses

**Fig. 2.**
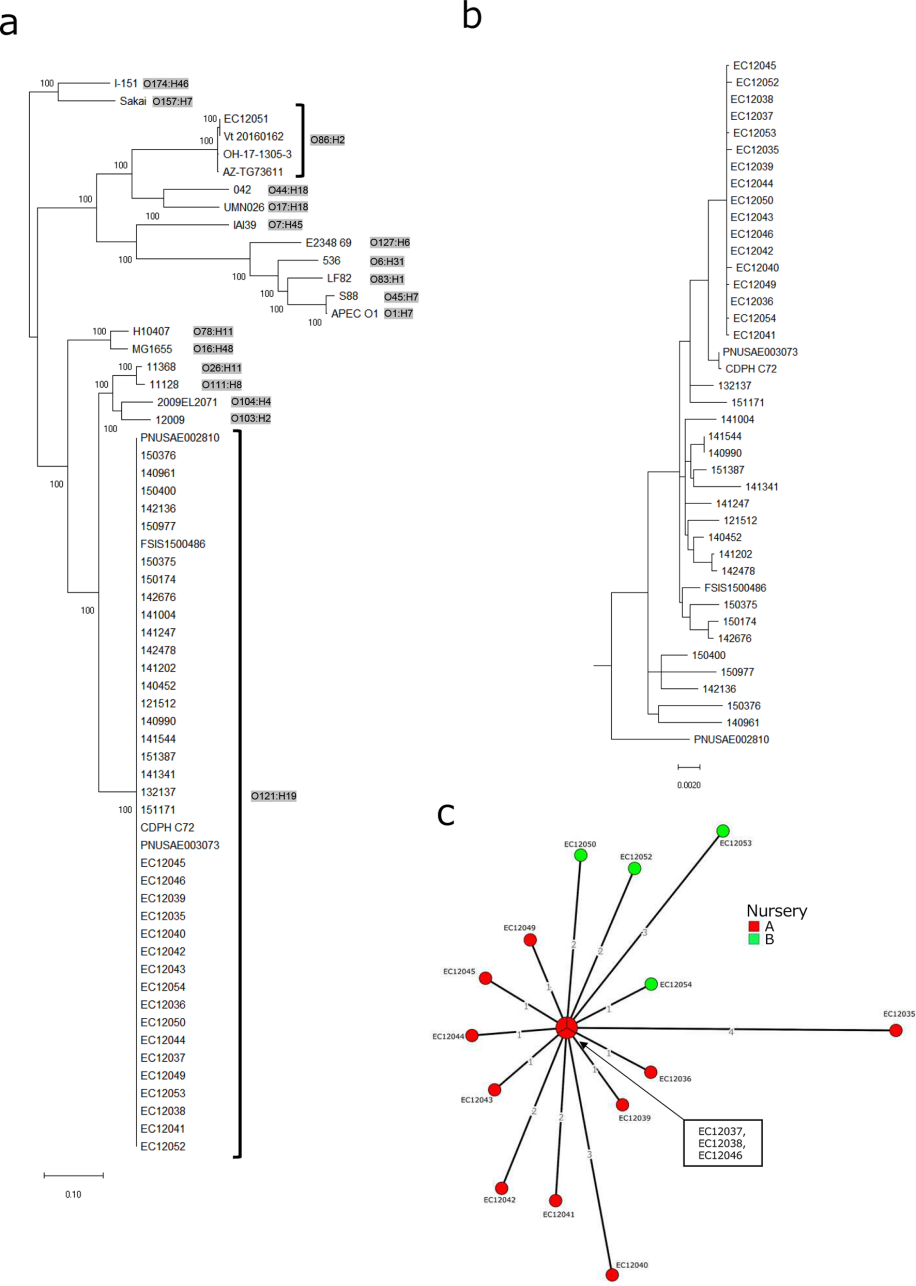
Phylogenetic analyses of the isolates. (a) Maximum-likelihood tree of the isolates from the nursery school outbreak and database strains. The scale bar represents the substitution rate per site. (b) Maximum-likelihood tree of EHEC O121. (c) Median joining tree of the nursery outbreak O121 isolates. The colours represent the nursery schools from which the isolates were obtained. The numbers of SNPs are shown on the connecting lines.

Core genome phylogenetic analysis revealed that the EHEC O121 isolates were genetically distant from the *E. coli* O86 isolate (Fig. 2a). The EHEC O121 isolates in this outbreak formed a cluster even in a subtree consisting solely of the O121 strains, suggesting the clonality of the isolates (Fig. 2b). A minimum spanning tree with these clustered isolates is shown in Fig. 2c. Three isolates had no detectable SNPs. The maximum number of SNPs between various isolates was seven. The isolate from the primary patient (EC12035) had the highest number of SNPs compared with the other isolates. According to *in silico* typing of the WGS contigs, all of the EHEC O121 isolates carried *stx2-* and LEE-encoding genes, including *eae* and *tir*, and this result was consistent with the PCR results. The *bla*_CTX-M-55_ sequence was detected in all of the ESBL-producing isolates, while no resistance gene was detected in the other O121 isolates. In contrast, the *E. coli* O86 isolate did not carry *stx-* or LEE-encoding virulence genes. The isolates carried various antimicrobial resistance genes, including *bla*_CTX-M-55_, *bla*_TEM-1C_, *aph(6)-Id*, *aph(3'')-Ib* and *sul2*.

### Determining plasmid sequences

**Fig. 3.**
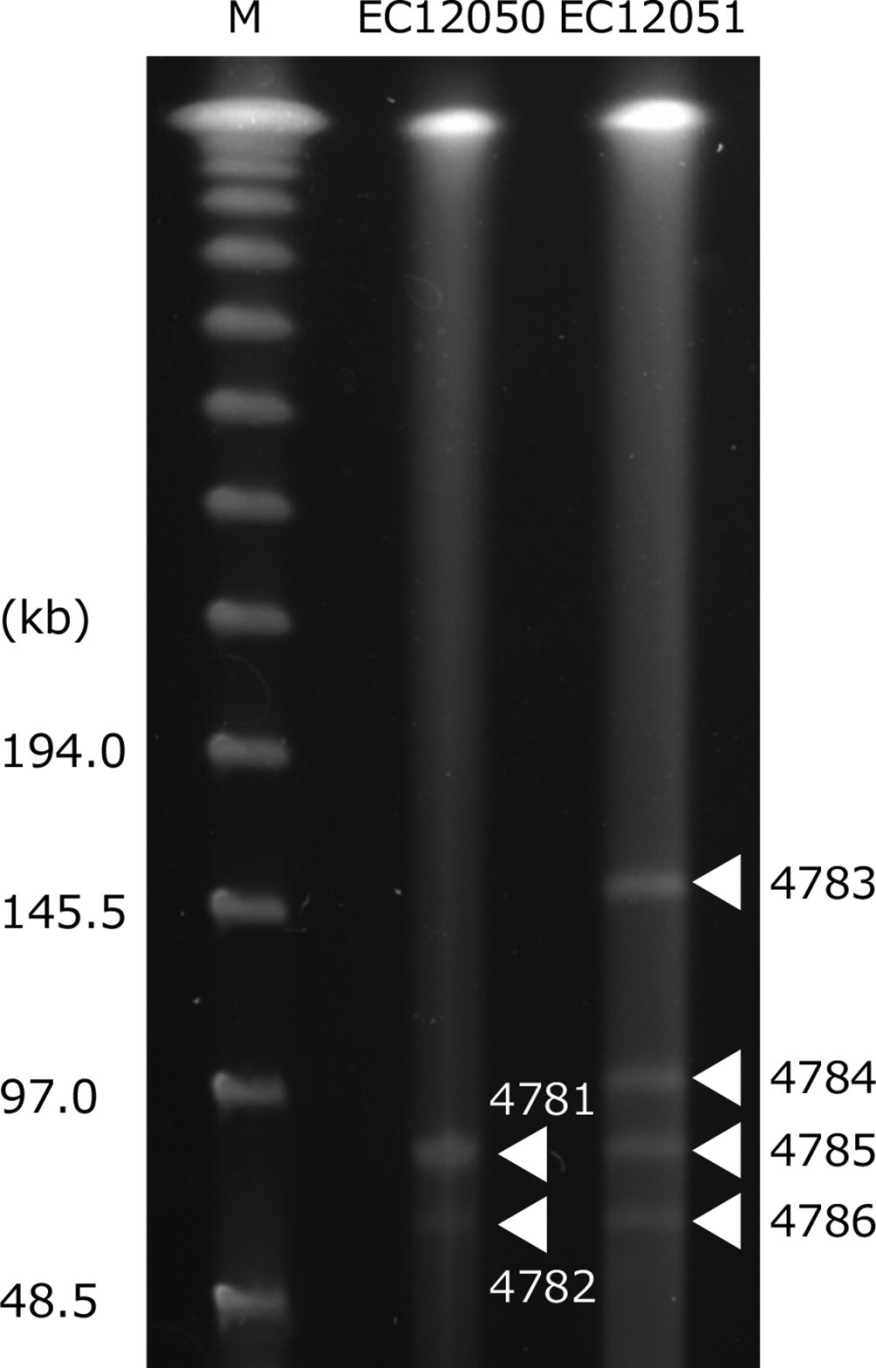
S1 PFGE image of enterohaemorrhagic *E. coli* O121 EC12050 and *E. coli* O86 EC12051. A Lambda PFG Ladder (New England Biolabs) was used as a marker. Open triangles indicate the bands that were analysed further.

In the S1-PFGE analysis, two bands (ca. 60 and 80 kbp) and four bands (ca. 60, 80, 90 and 150 kbp) were observed for EC12050 and EC12051, respectively (Fig. 3, Table 3). Short-read sequencing was performed with each band and the reads were assembled *de novo*. Because each band could be contaminated with other plasmid and chromosomal sequences, only contigs with high coverage (>×50) were used for further analyses. The total contig length was nearly in agreement with the estimated band size observed in the S1-PFGE, except for band 4781 of EC12050. This DNA band contained sequences that were highly similar to those of two plasmids from EHEC O121 2014–4423 (accession numbers: CP027455 and CP027456), whose sizes are 73 262 and 79 682 bp. Therefore, it was likely that band 4781 contained two plasmids. The other band of EC12050 carried the IncI2 plasmid sequence with *bla*_CTX-M-55_. *E. coli* O86 EC12051 carried two resistance plasmids. One plasmid, contained in band 4786, was IncI2 and harboured *bla*_CTX-M-55_ and was the ESBL plasmid of EHEC O121. The other plasmid, band 4784, carried *bla*_TEM-1C_, *aph(6)-Id, aph(3'')-Ib* and *sul2*. It was noted that nearly identical 61 kb contigs carrying the IncI2 replicon and *bla*_CTX-M-55_ were found in the EHEC O121 and *E. coli* O86 isolates (DNA bands 4782 and 4786). Therefore, we speculated that the contig was a plasmid and that EHEC O121 acquired the plasmid from *E. coli* O86. To verify the hypothesis, the complete sequence of the plasmid from EHEC O121 EC12050 was obtained. TA cloning was required to determine the complete sequence because the contig had a gap in a shufflon region. By TA cloning and subsequent Sanger sequencing, the complete sequence of the ESBL plasmid was determined, and this plasmid was designated pEC12050-CTX (DDBJ accession no. AP018926). The plasmid contained 61 613 bp and the sequence was highly similar (>99 % identity) to that of another *E. coli* ESBL plasmid (pHN1122-1, JN79750) and a *Shigella sonnei* plasmid (p1081-CTXM, KJ460501) isolated in China. Based on the short-read mapping, there were no SNPs or indels and thus the plasmid sequences in all of the EHEC O121 isolates and in *E. coli* O86 were likely to be highly similar or identical.

**Table 3.** Results of *in silico* analyses by sequencing S1-PFGE fragments

Isolate	Fragment	Estimatedband size (kb)	Total contiglength (bp)	Inc type	Resistance gene
EC12050(O121:H19)	4781	80	158 444	FIB, I1, B/O/K/Z	
	4782	60	67 134	I2	*bla*_CTX-M-55_
EC12051(O86:H2)	4783	150	150 659	FIC, FIB	
	4784	90	101 522	B/O/K/Z	*aph(6)-Id*, *aph(3'')-Ib*, *bla*_TEM-1C_, *sul2*
	4785	80	84 370	I1	
	4786	60	62 230	I2	*bla*_CTX-M-55_

### Conjugation assay

**Fig. 4.**
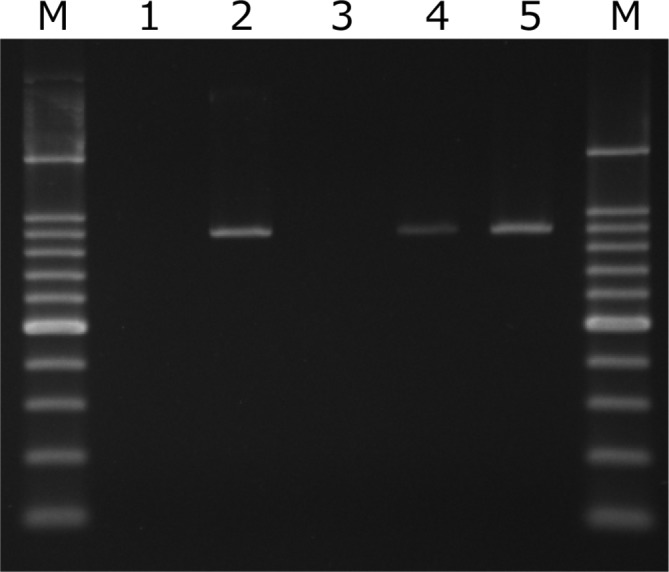
Electrophoresis gel image of PCR amplicons of CTX-M-1in ESBL plasmid-harbouring transconjugants. Lanes: M, marker, 100 bp DNA Ladder (Takara Bio, Inc.); 1, DH5α; 2, transconjugants (DH5α) selected by cefotaxime and rifampicin; 3, transconjugants selected by streptomycin and rifampicin;5, *E. coli* O121 EC12050; 6, *E. coli* O86 EC12051.

Transconjugants that were resistant to both cefotaxime and rifampicin were obtained in the conjugation assay. The frequency of transfer was 1.9×10^−2^. Five transconjugants were subjected to further analyses. The MICs of the antimicrobials for the five transconjugants increased to the same levels as those for the ESBL-producing EHEC O121 and *E. coli* O86 isolates (Table 2). We also confirmed that the transconjugants carried *bla*_CTX-M-55_ (Fig. 4). In a conjugation assay using streptomycin and rifampicin, transconjugants carrying *bla*_TEM-1c_ were obtained at a frequency of 3.7×10^−3^. These transconjugants were resistant to ampicillin but were susceptible to the other β-lactamases (Table 2).

## Discussion

In this outbreak, we confirmed the clonality of the EHEC O121 isolates from two nurseries using WGS analyses. These results also provided direct evidence for the transfer of the ESBL-producing plasmid to EHEC. These results demonstrate the importance of instructing nurseries in preventive measures, including training staff to take proper precautions against EHEC patients to control secondary infection as well as the spread of antimicrobial resistance.

Initially, we suspected a food-borne outbreak of EHEC. However, because the patients or carriers were reported for weeks, it is likely that some of the infections were due to secondary infection rather than to a single food source. WGS analysis supported the clonality of the EHEC O121 isolates. The isolate from the primary patient (EC12035) had the highest number of SNPs, suggesting that the EHEC O121 clone might have spread from the patient. In nursery A, the presence of a large number of asymptomatic carriers might have caused the larger outbreak through secondary infection. Because EHEC O121 was not detected in the kitchen staff or other staff members, we speculated that direct contact with faeces, such as during diaper changing, might have led to the secondary infection. Furthermore, joint care was held despite a request from the local health centre not to do so. These observations highlight the importance of early EHEC detection and implementation of control measures to prevent secondary infection by local health care authorities.

It was of note that the antimicrobial susceptibility profile of the EHEC O121 isolates changed during the development of this outbreak. According to the results of our sequence analysis and conjugation assays, it is plausible that EHEC O121 acquired pEC12050-CTX from *E. coli* O86 and subsequently spread in nursery B. Several outbreaks that were attributable to antimicrobial-resistant EHEC have been reported, including the involvement of AmpC *β*-lactamase-producing O157:H7 [33], CTX-M-15 ESBL-producing O111:H8 [34] and CTX-M-15 ESBL-producing O104:H4 [14]. However, to the best of our knowledge, there are currently few reports describing the acquisition of ESBL-producing plasmids during the development of an outbreak. This is probably the first report of EHEC isolates acquiring the ability to produce ESBL during the development of an outbreak.

In Japan, CTX-M-type enzymes are the most prevalent ESBLs [18, 31, 35]. CTX-M-55 is a variant of CTX-M-15 and has a single amino acid substitution at position 80 [36]. CTX-M-55 was first detected in Thailand, and since then *Enterobacteriaceae* isolates carrying *bla*_CTX-M-55_ have mainly been reported in Asian countries [36–38]. In Japan, EHEC strains harbouring ESBL have rarely been reported. Non-EHEC strains harbouring CTX-M-55 have been detected in animals [39–43], while there are few reports of their detection in humans [44]. As the Inc type of the resistance plasmids was unknown in most of the reports, the dissemination of an IncI2 plasmid carrying CTX-M-55 requires further study. The transmissibility of pEC12050-CTX and its potential to raise the MICs of antimicrobial agents could pose a risk to public health, and these features highlight the importance of monitoring the spread of this plasmid. Interestingly, conjugative transfer of the other resistance plasmid from *E. coli* O86, which harbours *bla*_TEM-1c_, was shown through *in vitro* conjugation assays. The frequency of transfer was comparable to that of pEC12050-CTX. It remains unclear why only the EHEC O121 isolate harbouring pEC12050-CTX, but not the other resistance plasmid, was disseminated in the outbreak, and this phenomenon requires further study.

The effect of ESBL production on the EHEC infection was unclear because the use of antimicrobials was avoided in this outbreak. The use of antimicrobials for treatment of EHEC infection remains controversial. It has been shown that antimicrobial treatment can enhance the development of HUS in EHEC-infected children [16, 45], while treatment using fosfomycin within the first 5 days of EHEC infection has been shown to be effective for mitigating the development of HUS in children [46] Although *in vitro* studies suggested that subinhibitory concentrations of antimicrobials, including *β*-lactams, can induce Stx production [47, 48], there is insufficient clinical evidence to draw a conclusion. Reliable clinical studies are required to elucidate the relationship between ESBLs and EHEC pathogenicity.

In conclusion, it is important to take appropriate control measures during EHEC outbreaks under the assumption that the causative strain can acquire antimicrobial resistance during the outbreak. EHEC outbreaks often occur in nurseries and elderly care facilities. People in these facilities are also likely to be susceptible to antimicrobial-resistant bacteria, including strains producing ESBLs and metallo-*β*-lactamases. Intensive surveillance and monitoring would be helpful to prevent the spread of EHEC as well as other antimicrobial-resistant bacteria in such facilities.

## Data bibliography

1. Kikuchi *et al.*, DDBJ, AP018926 (2018).

2. Kikuchi *et al.*, DDBJ, PRJDB7237 (2018).

## Supplementary Data

Supplementary File 1
