# Supplementary material for: Enterohaemorrhagic Escherichia coli O121:H19 acquired an extended-spectrum β-lactamase gene during the development of an outbreak in two nurseries
Source: Microb Genom. 2019 Jun 19;5(7):e000278. doi: 10.1099/mgen.0.000278 (PMC6700663; doi:10.1099/mgen.0.000278)
Supplement: Supplementary File 1 [file mgen-5-278-s001.pdf]

Table S1. Information of publicly available genomic data used in the phylogenetic analysis

ID	Serotype	Accession no.	Source	Year	Place
APEC_O1	O1:H7	CP000468	Poultry	NA	USA
12009	O103:H2	AP010958	Human	2001	Japan
2009EL-2071	O104:H4	SAMN01831190	Human	2009	Republic of Georgia
11128	O111:H8	AP010960	Human	2001	Japan
FSIS1500486	O121:H19	SAMN03922006	Livestock	2014	USA
PNUSAE003073	O121:H19	SAMN04999918	NA	NA	USA
CDPH_C72	O121:H19	SAMN05721788	NA	NA	USA
PNUSAE002810	O121:H19	SAMN04849837	NA	NA	USA
121512	O121:H19	SAMD00067399	Human	2008	Japan
132137	O121:H19	SAMD00067400	Human	2013	Japan
140452	O121:H19	SAMD00067401	Human	2014	Japan
140961	O121:H19	SAMD00067402	Human	2014	Japan
140990	O121:H19	SAMD00067403	Human	2014	Japan
141004	O121:H19	SAMD00067404	Human	2014	Japan
141202	O121:H19	SAMD00067405	Human	2014	Japan
141247	O121:H19	SAMD00067406	Human	2014	Japan
141341	O121:H19	SAMD00067407	Human	2014	Japan
141544	O121:H19	SAMD00067408	Human	2014	Japan
142136	O121:H19	SAMD00067409	Human	2014	Japan
142478	O121:H19	SAMD00067411	Human	2014	Japan
142676	O121:H19	SAMD00067412	Human	2014	Japan
150174	O121:H19	SAMD00067440	Human	2014	Japan
150375	O121:H19	SAMD00067441	Human	2014	Japan
150376	O121:H19	SAMD00067416	Human	2014	Japan
150400	O121:H19	SAMD00067432	Human	2014	Japan
150977	O121:H19	SAMD00067433	Human	2015	Japan
151171	O121:H19	SAMD00067437	Human	2015	Japan
151387	O121:H19	SAMD00067442	Human	2015	Japan
E2348_69	O127:H6	FM180568	Human	1969	UK
Sakai	O157:H7	BA000007	Human	1996	Japan
MG1655	O16:H48	CP025268	NA	NA	USA
UMN026	O17:H18	CU928163	Human	1998	USA
I-151	O174:H46	SAMN02732277	Human	2005	Germany
11368	O26:H11	AP010953	Human	2001	Japan
042	O44:H18	FN554766	Human	1983	Peru
S88	O45:H7	CU928161	Human	1989	France
536	O6:H31	CP000247	Human	1982	Germany
IAI39	O7:H45	CU928164	Human	1980	France
H10407	O78:H11	FN649414	Human	1973	Bangladesh
LF82	O83:H1	CU651637	Human	NA	France
Vt_20160162	O86:H2	SAMEA104188770	Human	NA	Vietnam
OH-17-1305-3	O86:H2	SAMN08975749	Poultry	2017	USA
AZ-TG73611	O86:H2	SAMN02628657	Poultry	2013	USA
